# Combating stigma in autism research through centering autistic voices: a co-interview guide for qualitative research

**DOI:** 10.3389/fpsyt.2023.1248247

**Published:** 2023-08-15

**Authors:** Elizabeth A. Kaplan-Kahn, Reid Caplan

**Affiliations:** ^1^Center for Autism Research, Children’s Hospital of Philadelphia, Philadelphia, PA, United States; ^2^Accessible Academia, Washington, DC, United States

**Keywords:** stigma, autism, qualitative, co-production, participatory research

## Abstract

As autism has gained increased attention in the past few decades, autistic advocates have adopted the phrase “Nothing about us without us,” illustrating the idea that autistic people should be centered in all conversations regarding autism. However, in a large portion of autism research, autistic people are still not meaningfully engaged throughout the research process, leading to continued stigma in research through biased methods. Thus, stigma about autism influences not only the content of autism research, but the ways in which neurotypical people conduct research alongside (or without) autistic people, ultimately resulting in less valid conclusions or research that actively harms the autistic community. One way to address this stigma is through involving autistic individuals as equal partners in the research process, such as by including autistic co-interviewers in qualitative studies of autistic people. In this perspectives piece, we will highlight the benefits of participatory research practices within qualitative research. Furthermore, we will outline methods for conducting co-interviews with autistic research partners and share insights from our experiences implementing this practice. We hope this piece provides researchers the practical resources and inspiration to continue working toward decreasing the stigma surrounding autism in research spaces.

## 1. Introduction

Over the past decade, the growing neurodiversity movement has undeniably impacted the landscape of autism research. However, autism stigma and biases against autistic people nevertheless continue to influence current empirical work. The impact of autism stigma and biases (whether conscious or unconscious) in research is pervasive and insidious in ways that create a harmful feedback loop – biases against autistic people influence study methodology, in turn impacting study framing, outcomes, and conclusions, which then leads to further entrenchment of autism stigma and bias. To break this cycle, non-autistic researchers must take intentional and explicit steps to identify places of potential bias in their research and take corrective action. Whether the research is quantitative or qualitative, it is imperative for researchers to recognize the hidden ways in which subjectivity creeps into research that we perceive and present as “objective” (1).

One way to begin to address stigma within autism research is increased autistic involvement in every phase of the research process. In this perspectives piece, we will first provide brief descriptions of qualitative studies centering the autistic experiences and community-based participatory methods in autism research. We will then integrate these two methodologies by discussing the benefits of having autistic people as equal contributors on qualitative interview teams. Next, we will outline methods for developing and conducting co-interviews with autistic researchers^1^ and non-autistic researchers, share insights from our experiences implementing this practice, and discuss some of the potential uses of this practice to reduce stigma against autistic people in autism research.

## 2. Centering autistic voices in autism research

Due to barriers that have historically and currently shut autistic people out of research spaces, many autism research teams are predominantly comprised of non-autistic researchers. The lack of autistic people on autism research teams poses a large risk of stigma against autism from the very inception of a research question. Put simply and generally, our lived experiences color and shape the questions and hypotheses we generate to learn more about autism. Non-autistic researchers, who have been trained extensively by other non-autistic professionals within the medical model of disability, undoubtedly have perspectives regarding autism that are biased by their experiences and include varying degrees of stigma about autism (2).

Addressing this stigma requires non-autistic researchers to put increased effort into providing opportunities for autistic people to share their perspectives and shape research agendas. One way to correct for this stigma is through an emphasis on qualitative research, in which autistic people are asked directly about their perspectives and lived experiences. The benefits of qualitative studies in autism research include gaining a deeper understanding of the autistic experience, developing more pertinent research questions, and deriving more accurate hypotheses (3). Suggestions and guidelines for conducting qualitative studies with autistic research participants are also available (4–6).

Literature on qualitative research and autism also includes recommendations to “involve stakeholders in some aspect of the research design and analysis” (6), emphasizing participatory methods in autism research. Comprehensive participatory research involves collaborating closely with autistic community members across multiple stages of research, including development, implementation, and dissemination (7, 8). Systematic reviews of such participatory research approaches in general healthcare research have established that such approaches offer a multitude of benefits (9–11). Applied to the field of autism research, these benefits can serve to decrease stigma in autism research, including closer alignment between the autism research and autistic communities (8), fostering novel and impactful programs of research (12), building trust between researchers and the autistic community (13) and conducting more ethical research practices in autism research (14).

As outlined above, we believe both qualitative studies and participatory methods independently serve important roles in decreasing autism stigma within autism research through centering autistic voices in both the research content and processes. We also posit that the combination of participatory methods and qualitative research can further decrease stigma and bias that can be present in autism research. Recent qualitative research exploring autistic adults’ experiences being interviewed by an autistic researcher revealed that participants felt increased connection and comfort during the interview process because they were speaking with another autistic individual (15). These approaches can have positive impacts on the science of autism, increasing not only the rigor and validity of autism research, but also its relevancy and impact.

In particular, we propose and recommend a co-interview procedure for conducting qualitative interviews with autistic participants, wherein at least one interviewer is an autistic researcher who is involved throughout the study process. There are the notable benefits of having an autistic researcher interview autistic participants (15, 16); however, co-interview approaches offer some practical and accessibility considerations that may make a co-interview approach more feasible for study teams. For example, currently, due to systemic barriers to research participation, there are a limited number of autistic researchers with the necessary training (e.g., the Collaborative Institutional Training Initiative – CITI training) to participate in institutional research. Further, the diversity of perspectives and neurotypes within the interview team further serves to decrease stigma within autism research through fostering connections between autistic and nonautistic researchers. Co-interview methods also promote opportunities for nonautistic researchers to examine their own stigma through working closely and collaboratively with autistic researchers during the interview process.

A researcher’s positionality (i.e., the researcher’s world view and relationship to the research study itself) influences the qualitative research process in many ways (17), and this premise is central to our recommendations. Our own positionalities as non-autistic (EKK) and autistic (RC) coauthors, as well as our identities as White people who communicate primarily through verbal speech, shape the current perspectives piece. We believe that having diverse positionalities on the research team and during interviews with participants benefits qualitative research in numerous ways, many of which serve to reduce prejudiced beliefs against autistic people that can lead to discrimination in autism research. These benefits occur at multiple points throughout the qualitative research process: prior to conducting interviews, during interviews, and after interviews are completed. Below, we outline these benefits while offering guidelines for developing and conducing co-interviews for qualitative studies in autism research (see Table 1 for Summaries of Guidelines and Benefits).

**Table 1:** Guidelines for co-interviews.

Timeline	Practice	Rationale
Before the interview	Interview protocol is written collaboratively with autistic researchers	Reduces chance of bias in interview questions* Increases understandability of interview questions for autistic participants* Increases content validity of the interview through ensuring that questions tap into the into the intended constructs*
	Co-interviewers discuss interview flow, including who will begin the interview and who will ask which questions	Establishes clear expectations for co-running the interview Minimizes risk of misunderstandings/miscommunications during the interview Promotes consistent interview procedures across participants
	Interview protocol and procedures are provided to participant prior to interview	Facilitates accessibility of interview by giving participants time to think about and respond to answers prior to start of interview* Decreases uncertainty of what participating in the interview will be like for the interviewee
During the interview	Co-interviewers introduce themselves to participant, stating relevant positionality for the interview	Provides autistic participant with relevant information about the positionality of the researchers* Increases knowledge equity in the interview – interviewee is provided with similar types of knowledge about interviewers, and they have about interviewee* Knowledge that there is an autistic person on the research team can lead to increased levels of comfort for participants and elicit more authentic responses*
	Co-interviewers actively listen to interview partner and participant responses when they are not the “active” interviewer	Ensures that interviewers are able to “step in” for each other at any point in the interview Promotes interview continuity between questions (e.g., referencing previous participant responses in a subsequent question)
	Co-interviewers provide clarification and re-wording of interview questions when uncertainty arises	Non-active interviewer may be able to re-word an interview question in a manner that is more accessible to the participant
	Co-interviewers are given opportunity to ask follow-ups to every interview question	Co-interviewers may bring different perspectives on important areas of insight in the participants’ answers* Provides opportunity for interviewers to clarify responses that may be potentially ambiguous in later data analysis
After the interview	Co-interviewers have dedicated time to debrief about and discuss the interview	Promotes opportunities for co-interviewers to provide feedback to each other* Co-interviewers given the opportunity to problem-solve any issues that arose during interview Encourages a mentality of open communication and feedback across all team members*
	Thematic analysis completed in collaboration with autistic and non-autistic researchers	Reduces chance of bias in interpretation of participant responses* Allows for novel autistic insights into interview themes*

Rationale with asterisks* indicate justifications that serve to reduce stigma in qualitative autism research.

## 3. Benefits of and guidelines for co-interview methods in qualitative autism research

Developing and implementing a protocol for co-interviews in qualitative autism research should be a collaborative practice at every stage. We highly recommend that before engaging in this type of work, all team members familiarize themselves with the principles of participatory methods in autism research (5, 8, 12, 13, 18) as well as best practices for qualitative research (19, 20). Here, we assume that the research teams have already developed their qualitative research ideas and questions. Additionally, we assume that these research ideas and questions have been developed collaboratively with autistic individuals.

The examples we provide are based on our team’s direct experiences of engaging in these processes. The goal of our study was to understand what daily living skills are most important to autistic adults, and how these daily living skills may or not be related to achieving their independence goals. Our study team included autistic and non-autistic researchers, as well as paid autistic consultants to review and provide feedback on our interview protocol. We (the co-authors of this piece) conducted all interviews with autistic participants.

### 3.1. Before the interview

Once study teams identify their primary research question, the next step is to collaboratively create the interview protocol. Questions for study teams to consider include how long the interview will be, how many questions are feasible in the allotted time, how to phrase questions to be maximally accessible for participants, and in what order questions would be most effective. Interview protocols should be drafted collaboratively with input from autistic and non-autistic researchers. This approach reduces the chance of unintentional, but potentially harmful, neurotypical biases in the interview questions.

For example, while developing the interview protocol, our research team engaged in multiple discussions around our conceptualization of “independence.” We discussed how best to communicate that our question was not to probe for participants’ levels of independence based on normative standards (i.e., living on one’s own without daily supports), but rather based on participants’ desired levels of independence (whatever those may be). Team members reflected on various ways that everyone receives support to live “independently” (e.g., hiring someone to do taxes, asking for help with home maintenance, etc.) and acknowledged that these supports change over time.

Including autistic researchers in the development of the interview protocol also increases the likelihood that the interview questions will be clear, understandable, and accessible to autistic participants (21). For example, while developing our daily living skills interview protocol, an autistic researcher suggested that asking participants to “describe a typical weekday” could be overwhelming or unclear for autistic participants without specifying an expected level of detail for the question. Relatedly, incorporating autistic perspectives into the creation of the interview protocol potentially enhances the content validity of the interview through ensuring that questions assess the intended constructs in autistic populations (22).

After the interview protocol is developed, co-interviewers should develop a plan for how interviews will be conducted. Aspects of the interview procedures that should be considered are who will begin the interview, which interviewer will ask each question, and how follow-up questions will be asked. We recommend that the interview procedure be written out for future reference. This practice establishes clear expectations for co-running the interview and minimizes the risk of miscommunications between the co-interviewers. Further, the process promotes consistent interview procedures across all participants. On our study team, we opted to alternate asking primary interview questions, ensuring that we each spent equal time in leadership and supportive roles.

To promote accessibility of the interview for participants, we recommend that the co-developed interview protocol and a description of interview procedures be provided to all participants prior to their interview. In our study, we provided participants a detailed description of the co-interview procedures, including brief co-interviewer biographies, instructions for the virtual interview platform, and communication options (e.g., video and audio, audio only, text chat). Providing descriptions of the interview procedure ahead of the study visit decreases uncertainty and ambiguity of what participating in the interview will be like, and allows participants take their time to process the questions and consider their responses. Further, providing co-interviewer biographies prior to the interview promotes transparency regarding the interviewer positionalities, allowing participants to consider their comfort level with the people they will be speaking with during the interview. If possible, we recommend that the interview questions and procedures be sent to participants at least 1 week in advance of the interview.

It should be noted that having two interviewers may increase the potential for participants to experience increased anxiety and/or sensory overload during the interview. Providing participants with the description of the interview procedures prior to the interview may help participants prepare for the interview experience and consider what accommodations they may find helpful ahead of the interview. For example, participants may request that all cameras are shut off during a virtual interview or may wear noise canceling headphones during in-person or virtual interviews to reduce sensory overload. Participants may also request to have a support person present during the interview to assist them as needed with social overwhelm or anxiety. In our experience, we have been able to make all accommodations that participants have requested, and this has led to increased rapport with participants.

### 3.2. During the interview

At the start of each interview, co-interviewers should introduce themselves to the participant, including any relevant positionality they want to share. For us, this practice included sharing our names, pronouns, institution/organization affiliation, and neurotype. Our goal in this practice was to increase equity in the types and levels of knowledge interviewees and interviewers had about each other. We believe that this introduction also implicitly communicated important team values to the participants – primarily that the study team valued neurodiversity and had taken explicit steps to attempt to decrease autism stigma and bias in our study.

Self-disclosure is a relatively common consideration in qualitative research (23), and can be a powerful tool for building rapport at the outset of the interview. Having an autistic researcher conduct interviews with participants communicated the team beliefs of valuing neurodiversity and autistic perspectives in research. Multiple participants made positive comments (e.g., “oh, that’s really cool”) when the autistic co-interviewer introduced themself at the start of the interview. Our hope is that this practice increased participants’ level of comfort and allowed them to provide more authentic responses, promoting the validity of their answers that were used in the subsequent thematic analyses.

Related to the sensory overload consideration noted above, we recommend that at the start of the interview, researchers re-iterate that there are multiple participation methods available to the participant and offer any available accommodations to reduce sensory overload. It should be made clear to participants that they can change their communication methods at any point during the interview. Some accommodation options available to participants during virtual interviews include (1) choosing to have their cameras off (i.e., audio only), (2) asking interviewers to have their cameras off (to reduce visual overload), and (3) using exclusively chat (i.e., no audio or visuals) to conduct the interview. Some accommodation options available to participants during in-person interviews include (1) wearing noise canceling headphones to reduce auditory overload, (2) allowing participants to determine the seating arrangement and distance in the interview room, and (3) asking participants how they would like to communicate their responses (i.e., spoken responses or written/typed responses). Pre-emptive steps can also be taken to reduce sensory overload for autistic participants. For example, in our study, only the co-interviewer who was actively asking the participant question would have their microphone un-muted. The other co-interviewer remained muted until it was time to move onto the next interview question.

We recommend that co-interviews listen attentively throughout the entire duration of the interview (i.e., during both questions they ask, and ones their co-interviewer asks). This practice ensures that either co-interviewer could step in to lead at any point in the interview and promotes interview continuity and clarity of interview questions. For example, we regularly referenced aspects of participants’ answers in subsequent interview questions. Further, during several interviews, we were able to provide clarification or re-wording for interview questions when the interviewee had questions about the item. Notably, different participants asked for clarification on different interview questions, and there was not a particular question (nor a particular interviewer) that interviewees found confusing. We both benefitted from each other’s perspectives when rewording questions to the participant, making the interview more understandable and accessible.

In addition to alternating primary interview questions, we developed a method in which the interviewer who asked the primary interview item also asked any follow-up questions to the item. After an interviewer felt that they were ready to move onto the next question, we explicitly gave our co-interviewer the opportunity to ask follow-up questions for the current question [e.g., “(NAME), do you have any follow-up questions before we move on?”]. The co-interviewer asked any additional follow-ups before moving to the next interview question. This procedure ensured that we were each given opportunities to address aspects of every question, while also making an explicit “hand-off” so that we both had clear understandings of who was taking the lead during the interview. We found this practice incredibly valuable, as each of us brought different perspectives on important areas of insight in participants’ responses. For example, on one occasion, our autistic co-interviewer (RC) asked a participant to clarify an important distinction about whether the participant’s response about their independence goals was about a goal they personally valued, or whether the response was driven by the goal being valued by others (i.e., participant’s parent). On another occasion, our non-autistic co-interviewer (EKK) asked a follow-up question that led the participant to share an important insight regarding the relationships between their levels of support, independence, and quality of life.

### 3.3. After the interview

In addition to the standard reflective practices regarding interview content after conducting a qualitative interview, co-interviewers may take time to reflect on the process of the interview after each participant. This practice gives the interviewers opportunities to provide feedback to each other and problem-solve and issues that may have arisen during the interview. For example, after experiencing technical/connectivity issues that resulted in poor audio-recording quality during an interview, we reflected on what types of technical challenges we could provide in-the-moment solutions for, and which we should opt to reschedule the interview. Our practice also gave us a space to reflect on ways that implicit bias or stigma may show up in our thoughts or actions, both personally and professionally. The space encouraged a mentality of open communication and feedback across team members that allowed for any mistakes to be pointed out, acknowledged, inspected, and corrected.

Collaboration with autistic and non-autistic researchers is also a crucial aspect of qualitative data analysis. Input from autistic researchers reduces that chance of neurotypical bias in interpreting participant responses. In our case, our team spent significant time reflecting on how normative views of what constitutes as “living independently” create stigma for autistic people who achieve desired levels of independence with various types of supports. Further, autistic perspectives allow for unique autistic insights into potentially novel themes in the interviews. For example, concepts like autistic burnout (24, 25), autistic inertia (26), and the double-empathy problem (27) were all introduced to the literature by autistic individuals.

## 4. Discussion

In this special issue on why stigma and bias surrounding autism are so detrimental to autistic people, we believe it is imperative to reflect how our own actions as researchers contribute to this problem. Research is necessary to establish strong evidenced-based ways to improve the lives of autistic people; however, autism research has undeniably caused harm to autistic people (1) and contributed to the levels of stigma and bias that autistic people continue to face today. Such experiences may lead people on the autism spectrum and their families to avoid participating in autism research altogether. This may be particularly true for autistic individuals with intersectional identities that face multiple types of stigmas, biases, and prejudices across personal and public levels (e.g., LGBTQIA+ autistic people, autistic people of color, autistic people from rural communities, non-speaking autistic people, etc.).

To help break this cycle, we believe it is essential for autism research to place an increased emphasis on centering autistic voices through qualitative research and participatory research methods. To our knowledge, this perspectives piece is the first to detail the benefits of and provide a guide for a co-interview approach to qualitative research. We believe that including diverse positionalities within the research team and during interviews with participants offers enormous benefit in decreasing potential bias and stigma in the research process while also increasing quality and rigor or qualitative science.

We recognize several considerations and limitations about our approach and experiences to date. First, as is true for other types of marginalized identities in research (28), it is important to consider the implications and potential burdens of encouraging people to identify themselves explicitly and publicly as autistic in positionality statements in autism research. Further efforts should be placed on creating environments to support autistic people interested in engaging in research and developing research cultures that address the concerns and needs of openly autistic researchers in an ongoing and transparent way.

Additionally, because this perspectives piece is written by two White researchers without intellectual disabilities who primarily use spoken language to communicate, we recognize that our recommendations likely contain biases that privilege certain kinds of knowledge and ways of communicating. Furthermore, our experience is limited to interviews with autistic participants who communicated primarily with verbal speech and did not have an intellectual disability. We acknowledge the important work that still needs to be done to include the voices of individuals – both researchers and participants – who are often left out of autism research, including, but not limited to, non-speaking autistics, autistic people with intellectual disability, autistic people of color, and autistic people with significant physical disabilities. We believe that additional qualitative work with these populations will be instrumental in progressing how research conceptualizes and studies topics that are priorities for these groups. We strongly urge researchers working in these spaces to adopt co-interviewing procedures when conducting their research.

We believe that the increased involvement of autistic people in autism research will help to reduce stigma surrounding autism in research spaces. While involvement can take many forms, in this guide, we provide rationale for and outline our practices of having autistic and non-autistic researchers co-interview autistic participants in a qualitative study. Our team included autistic and non-autistic researchers, as well as multiple paid autistic consultants who assisted us to develop our interview protocol. Based on these experiences, we found this collaborative approach reduced the chance of bias in interview questions, increased understandability of the interview questions for autistic participants, and provided multiple checks for our team to ensure our interview questions addressed our intended research questions. We hope this perspectives piece provides researchers the motivation, as well as some tangible steps, to reflect on the ways their research can benefit from the integration of autistic perspectives, both as qualitative research participants and as research collaborators.

## Data availability statement

The original contributions presented in the study are included in the article/supplementary material, further inquiries can be directed to the corresponding author.

## Author contributions

EKK and RC contributed to the conception of this perspectives piece. EKK wrote the first draft of the manuscript. All authors contributed to manuscript revision, read, and approved the submitted version.

## Funding

Funding for this article was provided by the Qualitative Methods Research Affinity Group at the Children’s Hospital of Philadelphia.

## Conflict of interest

Author RC is employed by company Accessible Academia.

The remaining authors declare that the research was conducted in the absence of any commercial or financial relationships that could be construed as a potential conflict of interest.

## Publisher’s note

All claims expressed in this article are solely those of the authors and do not necessarily represent those of their affiliated organizations, or those of the publisher, the editors and the reviewers. Any product that may be evaluated in this article, or claim that may be made by its manufacturer, is not guaranteed or endorsed by the publisher.

## Footnotes

^1^Here, we mean ‘autistic researcher’ to refer to any autistic person who is engaged in research practices, regardless of their education, training, degree, or employment.
